# A prospective cohort study of dietary indices and incidence of epithelial ovarian cancer

**DOI:** 10.1186/s13048-014-0112-4

**Published:** 2014-12-05

**Authors:** Jing Xie, Elizabeth M Poole, Kathryn L Terry, Teresa T Fung, Bernard A Rosner, Walter C Willett, Shelley S Tworoger

**Affiliations:** Channing Division of Network Medicine, Department of Medicine, Brigham and Women’s Hospital and Harvard Medical School, 181 Longwood Avenue, Boston, MA 02115 USA; Department of Epidemiology, Harvard School of Public Health, Boston, MA USA; Obstetrics and Gynecology Epidemiology Center, Department of Obstetrics and Gynecology, Brigham and Women’s Hospital and Harvard Medical School, Boston, MA USA; Department of Nutrition, Simmons College, Boston, MA USA; Department of Biostatistics, Harvard School of Public Health, Boston, MA USA; Department of Nutrition, Harvard School of Public Health, Boston, MA USA

**Keywords:** Alternative healthy eating index, Healthy eating index, Mediterranean diet score, Dietary pattern, Ovarian cancer, Prospective cohort, Epidemiology

## Abstract

**Background:**

Several dietary indices have been developed to measure overall diet quality, including the Healthy Eating Index-2005 (HEI-2005), which measures adherence to the 2005 Dietary Guidelines from the USDA; the Alternative Healthy Eating Index-2010 (AHEI-2010), which is based on foods and nutrients predictive of chronic disease risk; and the Alternate Mediterranean Diet Score (aMDS), which is an index that characterizes traditional food patterns of Mediterranean countries. Few studies have evaluated diet quality and ovarian cancer risk.

**Methods:**

We assessed the associations of the HEI-2005, AHEI-2010, and aMDS with risk of epithelial ovarian cancer prospectively among women in the Nurses’ Health Study. We used Cox proportional hazards models, adjusting for known ovarian cancer risk factors.

**Results:**

During 24 years of follow-up, we documented 696 incident epithelial ovarian cancer cases among 82,948 women with diet information. The multivariate adjusted hazard ratios (95% confidence interval; *P*trend) of epithelial ovarian cancer comparing the highest with the lowest quintile were 1.03 (0.80-1.34; 0.77) for the AHEI-2010, 0.85 (0.65-1.12; 0.57) for the HEI-2005, and 0.91 (0.71-1.18; 0.44) for the aMDS.

**Conclusions:**

We did not observe any clear association of three diet quality scores with ovarian cancer risk. Further work should other metrics of evaluating diet quality that may be more relevant cancer risk.

**Electronic supplementary material:**

The online version of this article (doi:10.1186/s13048-014-0112-4) contains supplementary material, which is available to authorized users.

## Introduction

Ovarian cancer is the fifth leading cause of cancer death for women in the U.S. [1]. There are substantial geographic differences in ovarian cancer incidence rates across the world, with the highest age-adjusted incidence rates in developed countries (greater than 10 per 100,000), except for Japan (6.4 per 100,000) [2]. One study reported that daughters of Japanese immigrants to the US had ovarian cancer incidence rates approaching the rates of American Caucasians [3]. This suggests that non-genetic and potentially modifiable risk factors, including dietary factors, may influence ovarian carcinogenesis. While ovarian cancer risk factors, including lack of oral contraceptive use, lack of tubal ligation, postmenopausal hormone therapy (HT), nulliparity, and a family history of breast or ovarian cancer [2], have been identified, few dietary factors have been consistently associated with the disease [4].

Prior studies of diet and ovarian cancer generally have evaluated only one food or nutrient at a time (e.g. [5-10]). Diet quality scores are based on *a priori* defined dietary patterns and are designed to evaluate an individual’s overall diet, usually derived from adherence to dietary guidelines. This measurement considers dietary intake as an overall pattern, rather than evaluating individual nutrients or food groups, which could lead to important information that may represent new pathways for prevention. While most diet quality scores were developed to predict cardiovascular outcomes [11,12], there is potential for these scores to predict other outcomes, such as ovarian cancer, that may have shared etiologic pathways like inflammation [13-15]. Only one study has evaluated a diet quality score and ovarian cancer risk in a population-based case–control study; no association was observed between the Healthy Eating Index 2005 (HEI-2005) with risk [16].

Using Food Frequency Questionnaire (FFQ) data collected from women in the Nurses’ Health Study (NHS), we prospectively examined the associations between three diet quality scores and risk of ovarian cancer. The diet-quality scores examined were the Alternative Healthy Eating Index 2010 (AHEI-2010), HEI-2005, and the Alternate Mediterranean Diet Score (aMDS).

## Methods

### Study population

The NHS is a prospective cohort initiated in 1976, when 121,700 female registered nurses, 30 to 55 years of age and residing in 11 U.S. states, completed an initial questionnaire. In 1984, we used a comprehensive FFQ to collect dietary data from participants. The cohort has been followed by biennially mailed questionnaires to update covariate information and ascertain non-fatal incident diseases. Deaths in the cohort are usually reported by family or postal authorities. We also search for names of non-responders in the National Death Index [17,18]. The follow-up rate through June 2010 of women who completed the 1984 FFQ was 97% of the potential person-years. Completion of the self-administered questionnaire was considered to imply informed consent. This analysis was approved by the institutional review board at the Brigham and Women’s Hospital.

### Dietary measurement and FFQ

Dietary information was measured by a self-administered semi-quantitative, 131-item FFQ, which was completed every 2 to 4 years in the cohort. For each food, a portion size was given and women were asked to choose from 9 intake frequencies, from never to ≥6 servings per day averaging over the prior year’s intake. Consumption of cold breakfast cereals, cooking oils, margarines, and multiple vitamins was specified by brand. The validity of our FFQ was evaluated by comparison with diet records in 192 NHS women; the average correlation for all foods was 0.63 [19-21].

### Diet-quality scores

The HEI-2005 measures adherence to the USDA Dietary Guidelines established in 2005, and is based on dietary recommendations to ensure adequate nutrient intake [22]; the components include total fruit, total vegetables, total grains, milk, and meat and beans (Additional file 1: Table S1). Additional components were created to represent whole fruit, dark green and orange vegetables and legumes, whole grains, and oils. The score ranges from 0 to 100. The HEI-2005 is a valid measure of diet quality in that the components are not strongly correlated with the overall score and that “exemplary” menus obtain a high score [23].

The AHEI-2010 was created as an alternative to the HEI; this index was designed to incorporate foods and nutrients that have been consistently associated with chronic disease risk [24]. It includes vegetables, fruits, whole grains, sugar-sweetened beverages (including fruit juices), nuts/legumes, red/processed meat, trans fat, long-chain omega-3 fatty acids, polyunsaturated fat, sodium, and alcohol (detailed in Additional file 1: Table S1); the score ranges from 0 to 110.

The aMDS is an index that characterizes traditional food patterns of Mediterranean countries. We used a modified version of the original MDS constructed by Trichopoulou and colleagues [25]. The aMDS considers consumption of certain fatty acids, legumes, cereals, fruits, nuts, vegetables, meat, dairy, and alcohol and ranges from 0 to 10 (Additional file 1: Table S1).

### Assessment of covariates

We collected extensive information on potential covariates, including demographic characteristics, body mass index (BMI), parity, oral contraceptive use, menopausal status, HT use, tubal ligation, caffeine intake, family history of ovarian cancer (in first degree family members), and other lifestyle factors. In general, most covariates were updated every 2–4 years.

### Identification of ovarian cancer cases

Ovarian cancer cases were identified either by self-report of the disease on a biennial questionnaire or through family members, the postal service, or the National Death Index. Women or their next of kin were asked for permission to obtain and review pathology reports to confirm the diagnosis and classify cancers by behavior (invasive vs. borderline), histologic type (e.g. epithelial vs. not; serous/poorly differentiated, endometrioid, clear cell, mucinous, other), and stage. For cases without medical records we attempted to confirm the diagnosis through the appropriate state cancer registry. We compared the pathology report histology with a review of slides by a gynecologic pathologist for 215 cases. The concordance was 98% for morphology and 83% for histology [26].

### Statistical analyses

At baseline we excluded women diagnosed with any cancer other than non-melanoma skin cancer (n = 8,458), women who reported a prior bilateral oophorectomy (n = 14,418), or menopause due to pelvic irradiation (n = 90). In addition, 1,448 NHS participants died before start of follow-up in 1986. Lastly, participants who did not complete the 1984 FFQ, had an implausible caloric intake in 1984 (<600 or >3,500 kcal/d), or left more than 70 items blank on the 1984 FFQ as well as participants who did not have dietary score data available in 1984 (n = 31,573) did not enter the analysis until they had valid dietary data. Participants accrued person-time from the return date of the 1986 questionnaire (or 2 years after the return of the first completed FFQ with plausible caloric intake and no more than 70 items blank) until the earliest of the following events: ovarian cancer diagnosis, any other cancer diagnosis (excluding non-melanoma skin cancer), bilateral oophorectomy, pelvic irradiation, death, or the end of follow-up (June 1, 2010).

Our primary exposure variables were cumulatively averaged diet scores (in quintiles) assessed at least 2 years before diagnosis (i.e., a 2-year lag) to account for potential influences of subclinical disease on dietary patterns. With this method, we used the diet score values from 1984 as the exposure for the follow-up period from 1986 to 1988, the average of the 1984 and 1986 values as the exposure for 1988 to 1992, the average of the 1984, 1986, and 1990 values as the exposure for 1992 to 1996, and so on. This approach allows the evaluation of long-term dietary intake with reduced measurement error, but maintaining true variations in diet [20]. We tested for linear trends using the medians of the quintile dietary scores as an ordinal variable.

We used Cox proportional hazards models, to estimate hazard ratios (HRs) and 95% confidence intervals (CIs) associated with quintiles of diet-quality scores. We handled time-varying covariates using the Andersen-Gill data structure [27], with covariate values set at the time the questionnaire was returned. To control for confounding by age, calendar time, and any 2-way interactions, we stratified jointly by age at the start of each follow-up period and calendar year of each follow-up period. The time scale was in months since the return date for each follow-up period. We tested proportional hazards assumptions using likelihood ratio tests comparing models with and without age (≤60, >60) interaction terms, p-values were 0.74, 0.97, and 0.88 for AHEI-2010, HEI-2005, and aMDS respectively.

For multivariate models, we adjusted for known and putative risk factors for ovarian cancer, including age (months), total energy intake (kcal/d), family history of ovarian cancer (yes, no), tubal ligation (yes, no), BMI (kg/m^2^), parity (yes, no), number of additional pregnancies (continuous), oral contraceptive use duration (0, >0-1 yrs, >1-5 yrs, and >5 yrs), smoking (pack-years), menopausal status (premenopausal/unknown, post-menopausal), type and duration of PMH use (never PMH user, past estrogen only user <5 yrs, past estrogen only user ≥5 yrs, current estrogen only user <5 yrs, current estrogen only user ≥5 yrs, past E + P user <5 yrs, past E + P user ≥5 yrs, current E + P user <5 yrs, and current E + P y user ≥5 yrs, past other user <5 yrs, past other user ≥5 yrs, current other user <5 yrs, and current other user ≥5 yrs,), age at menarche (years), hysterectomy (no, yes, unknown), unilateral oophorectomy (no, yes, unknown), lactose intake (g/d), caffeine intake (mg/d), and physical activity (0–3.8, >3.8-11.1, >11.1-26.4, >26.4 MET-hrs/wk, missing).

Secondarily, we evaluated the relations between dietary scores and specific subtypes of ovarian cancer using competing risk proportional hazards models [28], including invasive versus borderline, serous and poorly differentiated versus non-serous, and rapidly fatal (death from ovarian cancer within 3 years of diagnosis) versus less aggressive [29]. The estimates for the dietary scores, as well as tubal ligation, parity, and estrogen only HT use, were allowed to vary by subtype based on prior analyses, whereas estimates for the remaining covariates were constrained to a single effect estimate across subtypes. To test for heterogeneity by tumor subtype, we compared a model allowing the association for each dietary score to vary by subtype to a model that with the same association for all tumors. Analyses were conducted in SAS9.3 (SAS Institute, Cary, NC, USA) and all analyses used two-sided p-values.

## Results

We documented 696 incident epithelial ovarian cancer cases over 24 years of follow-up among 82,948 women. At the midpoint of follow-up (1998), women with a high AHEI-2010 score had lower BMI, higher physical activity, lower caffeine intake, higher lactose intake, and smoked less than those with lower scores (Table 1). Similar results were observed for the HEI-2005 and aMDS (Additional file 2: Table S2 and Additional file 3: Table S3). Based on the 1984 FFQ, the correlation was 0.63 (*P* < .0001) between HEI-2005 and AHEI-2010 scores, 0.43 (*P* < .0001) between HEI-2005 and aMDS, and 0.56 (*P* < .0001) between AHEI-2010 and aMDS.

**Table 1:** **Selected participant characteristics in 1998, the midpoint during follow-up, by quintiles of AHEI-2010 score among women in the NHS**

	**Quintiles of AHEI-2010 score**				
	**≤42 (N = 11255)**	**>42-48 (N = 12518)**	**>48-53 (N = 13169)**	**>53-59 (N = 13417)**	**>59 (N = 13273)**
Mean (SD)					
Age, years^*^	62.4 (7.1)	63.4 (7.1)	64.1 (7.1)	64.8 (7.1)	65.6 (7.0)
Age at menarche, years^*^	12.6 (1.4)	12.6 (1.4)	12.5 (1.4)	12.5 (1.4)	12.5 (1.4)
BMI, kg/m^2^	27.0 (5.5)	26.8 (5.2)	26.7 (5.2)	26.5 (5.0)	26.0 (4.9)
Years of OC use^a^	4.4 (3.8)	4.3 (3.8)	4.2 (3.9)	4.1 (3.9)	4.1 (4.0)
Parity^b^	3.3 (1.6)	3.3 (1.6)	3.2 (1.5)	3.2 (1.5)	3.1 (1.4)
Physical activity, MET-hr/wk	12.5 (17.4)	14.9 (18.3)	17.2 (21.6)	18.9 (21.6)	23.8 (27.0)
Lactose intake, mg/day	12.7 (9.6)	13.2 (9.1)	13.6 (9.2)	14 (9.3)	13.9 (9.0)
Caffeine intake, mg/day	287.9 (201.6)	291.6 (202.0)	285.5 (199.4)	278.9 (198.8)	259.7 (198.3)
Calories per day	1859 (451)	1777 (460)	1739 (465)	1705 (468)	1684 (461)
Pack-years of smoking^c^	31.8 (24.5)	27.6 (22.6)	25.3 (21.3)	22.7 (20.0)	20.5 (18.4)
E only HT use, years^d^	6.0 (5.6)	6.0 (5.7)	6.4 (6.0)	6.4 (5.8)	6.6 (5.9)
E + P HT use, years^e^	5.0 (3.2)	5.0 (3.3)	5.2 (3.3)	5.2 (3.4)	5.3 (3.5)
Other HT use, years^f^	3.0 (2.9)	2.9 (2.8)	3.0 (2.7)	3.0 (2.6)	3.1 (2.7)
Percent					
Ever OC use	49	49	51	51	52
Smoking status					
Never	47	45	44	42	40
Past	36	41	43	47	53
Current	17	14	12	10	7
Parous	95	95	95	94	93
Family history of ovarian cancer	3	3	3	3	3
Tubal ligation	21	21	21	21	21
Hysterectomy					
No	73	75	74	75	74
Yes	22	21	23	22	22
Unknown	5	4	3	3	3
Unilateral oophorectomy					
No	85	87	86	87	87
Yes	9	8	9	9	8
Unknown	6	5	4	4	4
Postmenopausal	92	92	93	93	92
Ever E only HT use	22	23	25	25	27
Ever E + P HT use	26	29	32	33	36
Ever other HT use	18	20	21	22	25

Values are standardized to the age distribution of the study population. Values of polytomous variables may not sum to 100% due to rounding.

^a^Among ever OC users.

^b^Among parous women.

^c^Among ever smokers.

^d^Among E only HT ever users.

^e^Among E + P HT ever users.

^f^Among other HT ever users.

^*^Value is not age adjusted.

Overall, the AHEI-2010 was not statistically significantly associated with epithelial ovarian cancer risk (*P*trend = 0.77). Compared to women with AHEI-2010 ≤ 42, the multivariate adjusted HRs (95% CI) were 1.02 (0.78-1.32) for AHEI-2010 >42-48, 1.23 (0.96-1.59) for AHEI-2010 >48-53, 1.11 (0.86-1.43) for AHEI-2010 > 53-59, and 1.03 (0.80-1.34) for AHEI-2010 > 59 (Table 2). The associations were similar by invasive versus borderline, serous/poorly differentiated versus non-serous, and rapidly fatal versus less aggressive tumors, (*P* heterogeneity = 0.91, 0.98, and 0.71, respectively) and when we restricted to women without a family history of ovarian cancer.

**Table 2:** **Hazard ratios (95% confidence intervals) for the association between 2-year lagged cumulative updated AHEI-2010 and ovarian cancer from 1986 through 2010 among women in the NHS**

		**AHEI-2010**					
	**N cases**	**≤42**	**>42-48**	**>48-53**	**>53-59**	**>59**	***P*** **trend**
# of cases		106	125	159	153	153	
Person-years		294,805	294,224	293,771	293,500	293,282	
Age-adjusted	696	1.00 (Ref)	1.04 (0.80,1.35)	1.29 (1.00,1.65)	1.17 (0.91,1.50)	1.12 (0.87,1.45)	0.30
Multivariable^*^	696	1.00 (Ref)	1.02 (0.78,1.32)	1.23 (0.96,1.59)	1.11 (0.86,1.43)	1.03 (0.80,1.34)	0.77
Invasive*	592	1.00 (Ref)	1.10 (0.83,1.46)	1.34 (1.02,1.76)	1.17 (0.88,1.54)	1.00 (0.75,1.34)	0.90
Borderline*	57	1.00 (Ref)	0.72 (0.32,1.67)	0.79 (0.34,1.83)	0.87 (0.39,1.95)	0.87 (0.38,1.95)	0.88
Serous/poorly differentiated*	435	1.00 (Ref)	1.19 (0.86,1.65)	1.32 (0.96,1.82)	1.17 (0.84,1.62)	0.99 (0.71,1.39)	0.69
Non-serous*	148	1.00 (Ref)	0.65 (0.37,1.12)	0.93 (0.56,1.53)	0.79 (0.47,1.33)	0.87 (0.53,1.44)	0.83
Rapidly fatal*	247	1.00 (Ref)	1.11 (0.72,1.70)	1.18 (0.78,1.80)	1.06 (0.70,1.63)	0.93 (0.61,1.44)	0.60
Less aggressive*	274	1.00 (Ref)	1.15 (0.76,1.74)	1.55 (1.05,2.29)	1.24 (0.82,1.86)	1.02 (0.66,1.55)	0.98

*Adjustment factors are listed in the statistical analyses section.

*P* values for heterogeneity by tumor subtype were 0.91, 0.98, and 0.71 for invasive versus borderline, serous/poorly differentiated versus non-serous, and rapidly fatal versus less aggressive tumors, respectively.

No clear associations were observed for the HEI-2005 (Table 3) or the aMDS (Table 4) and ovarian cancer risk or by tumor subtype. For example, women with HEI-2005 >74 versus ≤ 56 [i.e., top versus bottom quintile] had a HR of 0.85 (95% CI = 0.65-1.12, *P* trend = 0.57) and women with aMDS > 5.5 versus ≤2.6, the HR was 0.91 (95% CI = 0.71-1.18; *P* trend = 0.44). No heterogeneity was observed comparing different ovarian cancer subtypes for the HEI-2005 (*P* heterogeneity ≥ 0.32) or aMDS (*P* heterogeneity ≥ 0.41) and the results were similar when restricting to women without a family history of ovarian cancer.

**Table 3:** **Hazard ratios (95% confidence intervals) for the association between 2-year lagged cumulative updated HEI-2005 and ovarian cancer from 1986 through 2010 among women in the NHS**

		**HEI-2005**					
	**N cases**	**≤56**	**>56-63**	**>63-68**	**>68-74**	**>74**	***P*** **trend**
N cases		127	117	129	162	161	
Person-years		294,749	294,665	294,337	293,738	292,082	
Age-adjusted	696	1.00 (Ref)	0.82 (0.63,1.05)	0.85 (0.66,1.09)	0.99 (0.78,1.26)	0.92 (0.72,1.17)	0.98
Multivariable*	696	1.00 (Ref)	0.79 (0.61,1.03)	0.82 (0.63,1.06)	0.93 (0.72,1.20)	0.85 (0.65,1.12)	0.57
Invasive*	592	1.00 (Ref)	0.80 (0.60,1.05)	0.80 (0.60,1.05)	0.91 (0.69,1.20)	0.86 (0.64,1.15)	0.60
Borderline*	57	1.00 (Ref)	1.00 (0.45,2.24)	1.11 (0.50,2.43)	0.97 (0.42,2.20)	0.54 (0.20,1.43)	0.31
Serous/poorly differentiated*	435	1.00 (Ref)	0.81 (0.59,1.13)	0.91 (0.66,1.25)	0.91 (0.66,1.26)	0.90 (0.64,1.27)	0.79
Non-serous*	148	1.00 (Ref)	0.77 (0.46,1.31)	0.70 (0.41,1.21)	1.03 (0.62,1.69)	0.83 (0.49,1.43)	0.81
Rapidly fatal*	247	1.00 (Ref)	0.76 (0.51,1.14)	0.62 (0.40,0.94)	0.67 (0.44,1.02)	0.75 (0.50,1.13)	0.15
Less aggressive*	274	1.00 (Ref)	0.79 (0.52,1.18)	0.89 (0.60,1.32)	1.08 (0.73,1.59)	0.85 (0.556,1.29)	0.89

*Adjustment factors are listed in the Statistical Analyses section.

*P* values for heterogeneity by tumor subtype were 0.42, 0.95, and 0.32 for invasive versus borderline, serous/poorly differentiated versus non-serous, and rapidly fatal versus less aggressive tumors, respectively.

**Table 4:** **Hazard ratios (95% confidence intervals) for the association between 2-year lagged cumulative updated aMDS and ovarian cancer from 1986 through 2010 among women in the NHS**

		**aMDS**					
	**N cases**	**≤2.6**	**>2.6-3.5**	**>3.5-4.5**	**>4.5-5.5**	**>5.5**	***P*** **trend**
N cases		134	119	148	136	159	
Person-years		290,089	247,467	329,112	293,048	309,867	
Age-adjusted	696	1.00 (Ref)	0.91 (0.71,1.17)	0.85 (0.67,1.07)	0.86 (0.68,1.10)	0.93 (0.74,1.18)	0.52
Multivariable*	696	1.00 (Ref)	0.90 (0.70,1.16)	0.83 (0.65,1.05)	0.85 (0.66,1.09)	0.91 (0.71,1.18)	0.44
Invasive*	592	1.00 (Ref)	0.93 (0.71,1.22)	0.80 (0.61,1.03)	0.86 (0.66,1.13)	0.86 (0.65,1.13)	0.25
Borderline*	57	1.00 (Ref)	0.76 (0.33,1.78)	0.93 (0.44,1.94)	0.44 (0.17,1.15)	0.81 (0.37,1.78)	0.37
Serous/poorly differentiated*	435	1.00 (Ref)	0.95 (0.69,1.31)	0.93 (0.69,1.26)	0.86 (0.63,1.18)	0.87 (0.63,1.20)	0.29
Non-serous*	148	1.00 (Ref)	0.71 (0.42,1.19)	0.52 (0.31,0.88)	0.72 (0.44,1.20)	0.85 (0.53,1.38)	0.62
Rapidly fatal*	247	1.00 (Ref)	0.78 (0.51,1.18)	0.71 (0.48,1.06)	0.83 (0.56,1.23)	0.66 (0.44,1.00)	0.11
Less aggressive*	274	1.00 (Ref)	1.25 (0.85,1.84)	0.87 (0.59,1.29)	0.88 (0.59,1.32)	1.04 (0.70,1.55)	0.59

*Adjustment factors are listed in the Statistical Analyses section.

*P* values for heterogeneity by subtype were 0.63, 0.89, and 0.41 for invasive versus borderline, serous/poorly differentiated versus non-serous, and rapidly fatal versus less aggressive, respectively.

## Discussion

In this large prospective study, we did not observe any clear associations between three diet quality scores and the risk of epithelial ovarian cancer. Further, no association between the diet scores and risk were observed for specific ovarian cancer subtypes defined by invasiveness, histology, or tumor aggressiveness.

Our results are consistent with one small study of the HEI-2005 and ovarian cancer risk, which observed no association [16] and a prospective cohort study of the “Dietary Guidelines for Americans”, which also observed no association [30]. Studies using statistical approaches, such as principal components analysis, to assess dietary patterns in the population have not reported consistent associations with ovarian cancer, although there is some evidence that a pattern high in fat and meat may be associated with increased risk [31-34]. Other scores that include body size and physical activity in addition to diet factors inconsistently been associated with ovarian cancer risk, with no association in a European cohort [35] and a positive association in a US study [30]. The lack of association between these diet quality indices and ovarian cancer risk is also consistent with prior research that many of the individual components on the score, such as fruits, vegetables and alcohol, were not strongly associated with ovarian cancer risk, even in large pooled analyses [36,37].

The major strengths of our study are the large number of ovarian cancer cases; this enabled us to examine risk by tumor characteristics. We also had comprehensive information on diet and important covariates. All this information was collected prospectively, which reduces the potential for recall bias. In addition, dietary and covariate data were assessed multiple times throughout follow-up, which allows us to use long-term, cumulative average exposures and thus reduce within person variation. To reduce potential reverse causation by sub-clinical ovarian cancer influencing diet, we used a 2-year lagged analysis. Finally, the dietary scores calculated in our study have a wide distribution except for aMDS, suggesting that we have a good representation of different dietary habits in the US.

Several methodological issues should be considered in interpreting our findings. First, the diet scores are calculated using arbitrary weights that are assigned to each component, in part based on research on cardiovascular disease risk. Second, we cannot distinguish brands or processing methods for the food items on the FFQ, which may cause misclassification in nutrient content. Finally, self-reported diet assessed by FFQ has measurement error, even though we validated it with dietary records [21,38,39]. We expect the misclassification should be random with respect to the outcome and would lead to attenuation of associations.

Overall, in this large prospective cohort study among US women, we did not observe any clear associations with three scores of diet quality, AHEI-2010, HEI-2005, or aMDS, with the risk of epithelial ovarian cancer, suggesting that current dietary recommendations in the US may not influence ovarian cancer incidence. Given the limited number of ovarian cancer cases in individual cohorts, a pooled analysis of prospective cohorts should be considered to evaluate potential differential associations for more rare tumor subtypes and consideration of other dietary scores that better reflect factors associated with cancer is warranted.

## Additional files

Additional file 1: Table S1 Scoring criteria for AHEI-2010, HEI-2005, and aMDS. All scoring criteria are calculated per 1000 kilocalories/day unless specified, except saturated fat and SoFAAs, which are calculated as percentage of total energy. Polyunsaturated fat does not include EPA or DHA intake. For alcohol, the highest score was assigned to moderate, and the worst score to heavy, alcohol consumers. Nondrinkers received a score of 2.5. Additional file 2: Table S2 Selected participant characteristics in 1998, the midpoint during follow-up, by quintiles of HEI-2005 score among women in the NHS. Values are standardized to the age distribution of the study population. Values of polytomous variables may not sum to 100% due to rounding. a Among ever OC users. b Among parous women. c Among ever smokers. d Among E only HT ever users. e Among E + P HT ever users. f Among other HT ever users. * Value is not age adjusted. Additional file 3: Table S3 Selected participant characteristics in 1998, the midpoint during follow-up, by quintiles of aMDS score among women in the NHS. Values are standardized to the age distribution of the study population. Values of polytomous variables may not sum to 100% due to rounding. a Among ever OC users. b Among parous women. c Among ever smokers. d Among E only HT ever users. e Among E + P HT ever users. f Among other HT ever users. * Value is not age adjusted.

## Abbreviations

HEI-2005: Healthy Eating Index 2005
AHEI-2010: Alternative Healthy Eating Index 2010
aMDS: Alternate Mediterranean Diet Score
FFQ: Food frequency questionnaire
PMH: Postmenopausal hormone use
BMI: Body mass index
HR: Hazard ratio
CI: Confidence interval
