# Supplementary material for: A prospective cohort study of dietary indices and incidence of epithelial ovarian cancer
Source: J Ovarian Res. 2014 Dec 5;7:112. doi: 10.1186/s13048-014-0112-4 (PMC4263215; doi:10.1186/s13048-014-0112-4)
Supplement: Additional file 1: Table S1 — Scoring criteria for AHEI-2010, HEI-2005, and aMDS. All scoring criteria are calculated per 1000 kilocalories/day unless specified, except saturated fat and SoFAAs, which are calculated as percentage of total energy. Polyunsaturated fat does not include EPA or DHA intake. For alcohol, the highest score was assigned to moderate, and the worst score to heavy, alcohol consumers. Nondrinkers received a score of 2.5. [file 13048_2014_112_MOESM1_ESM.doc]

**Supplemental table 1. Scoring criteria for AHEI-2010, HEI-2005, and aMDS**

| **Alternative Healthy Eating Index 2010 (AHEI-2010)** | | |
| --- | --- | --- |
| ***Component*** | ***Points*** | ***Minimum to maximum score*** |
| *Vegetables* | *10* | *0 to ≥5 servings* |
| *Fruits* | *10* | *0 to ≥4 servings* |
| *Whole grains* | *10* | *Women: 0 to 75 g; men: 0 to 90 g* |
| *Sugar-sweetened beverages and fruit juice* | *10* | *≥1 servings/day to <1 serving per month* |
| *Nuts and legumes* | *10* | *0 to ≥1 serving* |
| *Red/processed meat* | *10* | *≥1.5 servings to <1 serving per month* |
| *Trans fat* | *10* | *≥4% to ≤0.5%* |
| *Long-chain omega-3 fats EPA+DHA* | *10* | *0 to ≥250 mg* |
| *Polyunsaturated fat* | *10* | *<2% to ≥10%* |
| *Sodium* | *10* | *Highest decile to lowest decile* |
| *Alcohol* | *10* | *Women: >2.5 to 0.5-1.5;*  *men: >3.5 to 0.5-2.0 drinks* |
| ***Total score*** | ***110*** |  |
| **Healthy Eating Index 2005 (HEI-2005)** | | |
| ***Component*** | ***Points*** | ***Minimum to maximum score*** |
| *Total Vegetables* | *5* | *0 to ≥1.1 cup* |
| *Dark Green & Orange Vegetables* | *5* | *0 to ≥0.4 cup* |
| *Total Fruit* | *5* | *0 to 0.8 cup* |
| *Whole Fruit (exclude juice)* | *5* | *0 to ≥0.4 cup* |
| *Total Grains* | *5* | *0 to ≥3.0 oz* |
| *Whole Grains* | *5* | *0 to ≥1.5 oz* |
| *Milk* | *10* | *0 to ≥1.3 cup* |
| *Meat and Beans* | *10* | *0 to ≥2.5 oz* |
| *Oils* | *10* | *0 to ≥12 g* |
| *Saturated Fat (% of energy)* | *10* | *≥15% to ≤7%* |
| *Sodium* | *10* | *Highest decile to lowest decile* |
| *Calories from Solid Fats, Alcoholic Beverages, Added Sugars (SoFAAs, %energy)* | *20* | *≥50%to ≤20%* |
| ***Total score*** | ***100*** |  |
| **Mediterranean Diet Score (aMDS)** | | |
| ***Component*** | ***Scoring criteria for 1 point, 0 point otherwise*** | |
| *Vegetables, serving/d* | *Greater than median intake* | |
| *Legume, serving/d* | *Same as above* | |
| *Fruit, serving/d* | *Same as above* | |
| *Nuts, serving/d* | *Same as above* | |
| *Whole grains, serving/d* | *Same as above* | |
| *Cereal fiber, serving/d* | *Same as above* | |
| *Fish, serving/d* | *Same as above* | |
| *Red and processed meat, serving/d* | *Less than median intake* | |
| *Ratio of monounsaturated to saturated fat, serving/d* | *Greater than median intake* | |
| *Alcohol, g/d* | *5-25* | |

All scoring criteria are calculated per 1000 kilocalories/day unless specified, except saturated fat and SoFAAs, which are calculated as percentage of total energy. Polyunsaturated fat does not include EPA or DHA intake. For alcohol, the highest score was assigned to moderate, and the worst score to heavy, alcohol consumers. Nondrinkers received a score of 2.5.
