# Supplementary material for: A prospective cohort study of dietary indices and incidence of epithelial ovarian cancer
Source: J Ovarian Res. 2014 Dec 5;7:112. doi: 10.1186/s13048-014-0112-4 (PMC4263215; doi:10.1186/s13048-014-0112-4)
Supplement: Additional file 2: Table S2 — Selected participant characteristics in 1998, the midpoint during follow-up, by quintiles of HEI-2005 score among women in the NHS. Values are standardized to the age distribution of the study population. Values of polytomous variables may not sum to 100% due to rounding. a Among ever OC users. b Among parous women. c Among ever smokers. d Among E only HT ever users. e Among E + P HT ever users. f Among other HT ever users. * Value is not age adjusted. [file 13048_2014_112_MOESM2_ESM.doc]

**Supplementary table 2. Selected participant characteristics in 1998, the midpoint during follow-up, by quintiles of HEI-2005** score among women in the NHS

|  | **Quintiles of HEI-2005 score** | | | | |
| --- | --- | --- | --- | --- | --- |
|  | ≤56  (N=10796) | >56-63  (N=12584) | >63-68  (N=13491) | >68-74  (N=13689) | >74  (N=13071) |
| Mean (SD) |  |  |  |  |  |
| Age, years^*^ | 62.6 (7.0) | 63.2 (7.1) | 64.0 (7.1) | 64.7 (7.1) | 65.9 (7.0) |
| Age at menarche, years^*^ | 12.7 (1.4) | 12.6 (1.4) | 12.5 (1.4) | 12.5 (1.4) | 12.5 (1.4) |
| BMI, kg/m² | 26.6 (5.5) | 26.8 (5.3) | 26.7 (5.1) | 26.6 (4.9) | 26.3 (4.9) |
| Years of OC use^a^ | 4.5 (3.9) | 4.2 (3.8) | 4.2 (3.8) | 4.2 (3.9) | 4.0 (3.9) |
| Parity^b^ | 3.3 (1.7) | 3.2 (1.6) | 3.2 (1.5) | 3.2 (1.5) | 3.0 (1.4) |
| Physical activity,  MET-hr/wk | 13.2 (18.6) | 15.4 (19.5) | 17.0 (20.2) | 19.2 (22.8) | 22.5 (25.8) |
| Lactose intake, mg/day | 9.6 (8.0) | 11.4 (8.0) | 13.1 (8.4) | 14.7 (8.9) | 17.9 (10.3) |
| Caffeine intake, mg/day | 331.9 (211.3) | 300.1 (199.0) | 283.0 (194.3) | 262.8 (193.2) | 230.8 (190.4) |
| Calories per day | 1938 (508) | 1839 (465) | 1764 (448) | 1689 (424) | 1560 (402) |
| Pack-years of smoking^c^ | 34.9 (24.9) | 27.4 (22.5) | 23.8 (20.0) | 21.4 (19.3) | 19.2 (17.7) |
| E only HT use, years^d^ | 6.0 (5.7) | 6.0 (5.7) | 6.3 (5.7) | 6.6 (5.9) | 6.5 (5.9) |
| E+P HT use, years^e^ | 5.0 (3.2) | 5.1 (3.3) | 5.2 (3.3) | 5.3 (3.4) | 5.3 (3.4) |
| Other HT use, years^f^ | 2.9 (2.8) | 2.8 (2.7) | 3.0 (2.7) | 3.1 (2.9) | 3.1 (2.6) |
| Percent |  |  |  |  |  |
| Ever OC use | 49 | 51 | 51 | 51 | 51 |
| Smoking status |  |  |  |  |  |
| Never | 36 | 43 | 45 | 46 | 48 |
| Past | 40 | 43 | 45 | 46 | 47 |
| Current | 24 | 14 | 10 | 8 | 5 |
| Parous | 95 | 95 | 95 | 95 | 94 |
| Family history of ovarian cancer | 3 | 3 | 3 | 3 | 3 |
| Tubal ligation | 20 | 22 | 21 | 21 | 20 |
| Hysterectomy |  |  |  |  |  |
| No | 73 | 75 | 75 | 74 | 74 |
| Yes | 21 | 21 | 22 | 23 | 22 |
| Unknown | 6 | 4 | 3 | 3 | 3 |
| Unilateral oophorectomy |  |  |  |  |  |
| No | 84 | 86 | 87 | 87 | 87 |
| Yes | 9 | 9 | 9 | 9 | 9 |
| Unknown | 7 | 5 | 4 | 4 | 4 |
| Postmenopausal | 93 | 92 | 92 | 92 | 93 |
| Ever E only HT use | 22 | 24 | 25 | 27 | 26 |
| Ever E+P HT use | 25 | 30 | 32 | 34 | 35 |
| Ever other HT use | 18 | 20 | 21 | 23 | 25 |

Values are standardized to the age distribution of the study population. Values of polytomous variables may not sum to 100% due to rounding.

^a^ Among ever OC users

^b^ Among parous women

^c^ Among ever smokers.

^d^ Among E only HT ever users

^e^ Among E+P HT ever users

^f^ Among other HT ever users

^*^ Value is not age adjusted
