# Supplementary material for: A prospective cohort study of dietary indices and incidence of epithelial ovarian cancer
Source: J Ovarian Res. 2014 Dec 5;7:112. doi: 10.1186/s13048-014-0112-4 (PMC4263215; doi:10.1186/s13048-014-0112-4)
Supplement: Additional file 3: Table S3 — Selected participant characteristics in 1998, the midpoint during follow-up, by quintiles of aMDS score among women in the NHS. Values are standardized to the age distribution of the study population. Values of polytomous variables may not sum to 100% due to rounding. a Among ever OC users. b Among parous women. c Among ever smokers. d Among E only HT ever users. e Among E + P HT ever users. f Among other HT ever users. * Value is not age adjusted. [file 13048_2014_112_MOESM3_ESM.doc]

**Supplementary table 3. Selected participant characteristics in 1998, the midpoint during follow-up, by quintiles of aMDS** score among women in the NHS

|  | **Quintiles of aMDS score** | | | | |
| --- | --- | --- | --- | --- | --- |
|  | ≤2.6  (N=11953) | >2.6-3.5  (N=10260) | >3.5-4.5  (N=14451) | >4.5-5.5  (N=13048) | >5.5  (N=13920) |
| Mean (SD) |  |  |  |  |  |
| Age, years^*^ | 62.4 (7.1) | 63.4 (7.1) | 64.1 (7.1) | 64.8 (7.2) | 65.5 (7.0) |
| Age at menarche, years^*^ | 12.6 (1.4) | 12.5 (1.4) | 12.5 (1.4) | 12.5 (1.4) | 12.6 (1.4) |
| BMI, kg/m² | 26.9 (5.4) | 26.8 (5.2) | 26.7 (5.2) | 26.6 (5.1) | 26.1 (4.9) |
| Years of OC use^a^ | 4.4 (3.7) | 4.3 (3.8) | 4.2 (3.8) | 4.1 (3.9) | 4.1 (4.0) |
| Parity^b^ | 3.2 (1.6) | 3.2 (1.6) | 3.2 (1.5) | 3.2 (1.5) | 3.1 (1.5) |
| Physical activity,  MET-hr/wk | 12.4 (17.5) | 14.8 (20.1) | 16.3 (19.7) | 19.3 (22.4) | 23.9 (26.0) |
| Lactose intake, mg/day | 14.3 (11.3) | 13.7 (9.9) | 13.5 (9.0) | 13.3 (8.5) | 12.9 (7.7) |
| Caffeine intake, mg/day | 329.4 (231.3) | 302.2 (210.6) | 283.2 (196.8) | 263.0 (182.3) | 232.1 (167.1) |
| Calories per day | 1513 (418) | 1611 (425) | 1720 (436) | 1841 (445) | 2004 (449) |
| Pack-years of smoking^c^ | 31.6 (24.0) | 28.0 (22.4) | 25.5 (21.6) | 22.7 (20.2) | 19.4 (17.9) |
| E only HT use, years^d^ | 6.1 (5.8) | 6.1 (5.6) | 6.3 (5.8) | 6.4 (5.8) | 6.6 (6.0) |
| E+P HT use, years^e^ | 5.1 (3.2) | 5.1 (3.2) | 5.2 (3.4) | 5.3 (3.4) | 5.3 (3.5) |
| Other HT use, years^f^ | 3.0 (3.0) | 2.9 (2.5) | 2.9 (2.6) | 3.0 (2.7) | 3.1 (2.8) |
| Percent |  |  |  |  |  |
| Ever OC use | 49 | 51 | 51 | 50 | 52 |
| Smoking status |  |  |  |  |  |
| Never | 41 | 43 | 44 | 45 | 45 |
| Past | 40 | 43 | 44 | 46 | 48 |
| Current | 19 | 15 | 12 | 9 | 7 |
| Parous | 95 | 95 | 95 | 94 | 94 |
| Family history of ovarian cancer | 3 | 3 | 3 | 3 | 3 |
| Tubal ligation | 21 | 21 | 21 | 21 | 20 |
| Hysterectomy |  |  |  |  |  |
| No | 74 | 74 | 74 | 74 | 75 |
| Yes | 21 | 22 | 22 | 23 | 22 |
| Unknown | 5 | 4 | 4 | 3 | 3 |
| Unilateral oophorectomy |  |  |  |  |  |
| No | 86 | 86 | 87 | 87 | 87 |
| Yes | 8 | 9 | 9 | 9 | 8 |
| Unknown | 6 | 5 | 4 | 4 | 4 |
| Postmenopausal | 93 | 92 | 92 | 93 | 93 |
| Ever E only HT use | 22 | 24 | 26 | 26 | 26 |
| Ever E+P HT use | 27 | 30 | 31 | 33 | 35 |
| Ever other HT use | 19 | 20 | 21 | 22 | 24 |

Values are standardized to the age distribution of the study population. Values of polytomous variables may not sum to 100% due to rounding.

^a^ Among ever OC users

^b^ Among parous women

^c^ Among ever smokers.

^d^ Among E only HT ever users

^e^ Among E+P HT ever users

^f^ Among other HT ever users

^*^ Value is not age adjusted
